# Supplementary material for: Adoption and validation of the European Association for the Study of the Liver algorithm for the noninvasive diagnosis of advanced fibrosis in metabolic dysfunction-associated steatotic liver disease in low-resource South Asian settings
Source: PLoS One. 2026 Feb 6;21(2):e0341364. doi: 10.1371/journal.pone.0341364 (PMC12880717; doi:10.1371/journal.pone.0341364)
Supplement: Attachment — Submitted filename: Responses to reviewer comments_R1.docx [file pone.0341364.s001.docx]

**Title: Adoption and validation of the European Association for the Study of the Liver algorithm for the noninvasive diagnosis of advanced fibrosis in metabolic dysfunction-associated steatotic liver disease in low-resource South Asian settings.**

**Response to reviewer comments:**

1. Comment: In table 1, complete all parentheses

Response: Thank you for picking it. We corrected all.

2. Comment: In table I, use the universal recognized categories for BMI according to cut-off values. You may need to recalculate the frequencies. Thank you.

Response: We recalculated and included it.

3. Comment: In table I, divide the waist by sex and treat it as a numeric discrete variable. Waist itself is related to liver steatosis and could be a great prognosis factor to MASLD, however it requieres a better statistical treatment, I suggets that you could consider it.

Response: Thank you for the constructive comment. In the baseline characteristics, we demonstrated abdominal obesity, as classified universally, in both men and women. Now, in the revised version, we have shown the mean waist in men and women separately, as you rightly pointed out. It was normally distributed.

4. Comment:Explain the importance to divide by ethnic groups, if there is no another analysis with this variable, is enough to stablish "Sinhalese" and "other ethnicities" (Tamil, Muslim, Burgher, etc.).

Response: Thank you. Agree with your comment. We do see a lot of Muslim people having steatotic liver disease, but when we separately analysed ethnicity as an associated factor for SF, it did not show any significant association. Probably it is due to Muslim people having high BMIs. So changed the ethnicity as you suggested.

5. Comment: Describe the reasons why 7 participants were lost.

Response: These patients did not attend the fibroscanning appointment despite receiving reminders.

6. Comment: In the discussion section, please state the real utility of your new criterion for VCTE using different frequencies of the disease, and please give examples of the likelihood ratio.

Response: Thank you for the comment to improve the paper. We calculated positive/negative likelihood ratios (lines 234-237) and strengthened our discussion using these results (lines 269-273).
